# Fish oil-containing lipid emulsions prevention on parenteral nutrition-associated cholestasis in very low birth weight infants: a meta-analysis

**DOI:** 10.1007/s12519-022-00536-2

**Published:** 2022-03-24

**Authors:** Ting-Ting Zou, Jin-Rong Li, Yu Zhu, Chao-Min Wan, Qiong Liao

**Affiliations:** 1 Department of Pediatric Infectious Diseases, Key Laboratory of Birth Defects and Related Diseases of Women and Children, Ministry of Education, West China Second University Hospital, Sichuan University, Chengdu, 610041 China; 2 Department of Child Healthcare, Key Laboratory of Birth Defects and Related Diseases of Women and Children, Ministry of Education, West China Second University Hospital, Sichuan University, Chengdu, 610041 China

**Keywords:** Extremely low birth weight infant, Fish oil, Lipid emulsion, Parenteral nutrition-associated cholestasis, Very low birth weight infant

## Abstract

**Background:**

The effect of fish oil-containing lipid emulsions on preventing parenteral nutrition-associated cholestasis (PNAC) in very low birth weight (VLBW) infants is not known. Thus, we conducted a meta-analysis to identify any prevention effect.

**Methods:**

PubMed, EMBASE, and CENTRAL were searched up to 26 January 2021 for studies related to the preventive effect of fish oil-containing lipid emulsions and fish oil-free lipid emulsions on cholestasis in VLBW infants. Revman 5.3 was used to synthesize the results. A fixed-effect model was used to summarize the data when the heterogeneity was non-significant (*I*^2^ < 50%), and a random-effects model was used when the heterogeneity was significant (*I*^2^ > 50%).

**Results:**

Of 728 articles, 11 randomized controlled trials met the inclusion criteria. The meta-analysis indicated that fish oil-containing lipid emulsion reduced the occurrence of PNAC significantly with risk ratio (RR) = 0.53, 95% confidence interval (CI) 0.36–0.80, *P* = 0.002. The heterogeneity was non-significant with *I*^2^ = 23%. Subgroup analysis based on parenteral nutrition duration and median birth weight was performed. The synthesis results for patients with parenteral nutrition duration exceeding 14 days revealed *I*^2^ = 35% (*P* = 0.15) and pooled RR = 0.47, 95% CI 0.30–0.73, *P* = 0.0008; and for patients with duration less than 14 days revealed *I*^2^ = 0% (*P* = 0.72) and pooled RR = 1.14, 95% CI 0.39–3.35, *P* = 0.81. The synthesis for patients with birth weight more than 1000 g revealed *I*^2^ = 0% (*P* = 0.41) and pooled RR = 0.55, 95% CI 0.26–1.18, *P* = 0.12; and for patients with birth weight below 1000 g revealed *I*^2^ = 44% (*P* = 0.11) and pooled RR = 0.53, 95% CI 0.33–0.85, *P* = 0.009.

**Conclusions:**

The fish oil-containing lipid emulsion can reduce the occurrence of PNAC in VLBW infants based on the available original randomized controlled trial studies, especially for patients with parenteral nutrition duration exceeding 14 days and extremely low birth weight infants. Future studies should be performed before a definitive conclusion can be established.

## Introduction

Infants born with birth weight less than 1500 g are defined as very low birth weight (VLBW) infants. Among them, infants born with birth weight less than 1000 g are defined as extremely low birth weight (ELBW) infants. With the establishment of neonatal intensive care units and the development of medical technology, including mechanical ventilators and artificial pulmonary surfactant utilization, the survival rate of VLBW infants has increased in recent years, and is currently 80.6–93.8% according to different regions [1–5]. Therefore, the growth and development of VLBW infants has drawn much attention from physicians. One major remaining problem is nutrition supplementation.

Usually, enteral nutrition is not feasible immediately after birth for VLBW infants, because they are susceptible to respiratory distress syndrome, hypotension, temperature instability, and gut immaturity. Thus, parenteral nutrition (PN) should be initiated as soon as possible after birth of VLBW infants to avoid growth failure in postnatal life [6]. However, PN may have short- and long-term adverse effects in VLBW infants. Cholestasis is the main complication related to long-term use of PN, with an incidence range of 15.7–60.9% depending on the duration [7]. Cholestasis can develop into irreversible inflammatory/fibro-cirrhotic liver disease if not properly recognized and treated [8]. Parenteral nutrition-associated cholestasis (PNAC) is the clinical condition in which cholestasis results from prolonged PN, and that is otherwise unexplained. The occurrence of PNAC is related to prematurity, low birth weight, and intrauterine growth restriction, indicating that liver immaturity is the main risk factor of PNAC [9]. Previous studies demonstrated that PNAC risk is highest among infants with birth weight below 1500 g [10, 11]. Prevention of PNAC is important to improve the survival rate and prognosis of VLBW infants.

The mechanism of PNAC is not established and seems to be multifactorial. Some evidence suggests that high content of ω-6 polyunsaturated long-chain fatty acids in soybean oil may exert pro-inflammatory effects and accumulation of phytosterols can reduce bile flow, both of which are linked to development of PNAC [12, 13]. An increasing number of studies have found that fish oil-containing lipid emulsions, rich in ω-3 long-chain polyunsaturated fatty acids, may limit the level of phytosterols and regulate lipid and glucose metabolism to reduce the liver injury and reduce occurrence of PNAC in animal model and in neonates [14–17].

Previous meta-analysis demonstrated that fish oil-containing lipid emulsion can prevent PNAC in neonates [18]. However, the included studies rarely focused on VLBW infants. Several more recent original studies have focused on the influence of fish oil-containing emulsions on PNAC of VLBW neonates. As a single-center result is not convincing, we conducted a meta-analysis to produce higher grade evidence.

## Methods

We performed the meta-analysis according to PRISMA (Preferred Reporting Items for Systematic Reviews and Meta-Analyses) guidelines. The study was registered on INPLASY. The registration number is INPLASY2021110046. The protocol was available on the INPLASY website.

### Databases and search strategy

We searched PubMed, EMBASE, and CENTRAL up to 26 January 2021 using the search terms “very low birth weight infant? OR VLBW OR extremely low birth weight infant? OR ELBW OR low birth weight infant? OR preterm infant? OR prematurity OR premature?” and “fish oils OR fatty acids, omega-3 OR eicosapentaenoic acid OR docosahexaenoic acids OR fish-oil lipid emulsion? OR (fish? and oil?) OR (fish? adj2 lipid?) OR (fish? adj2 emulsion?) OR (fish? adj2 fat?) OR (omega-3 adj2 fatty acid?) OR eicosapentaenoic acid? OR icosapentaenoic acid? OR docosahexaenoic acid? OR SMOF OR SMOF-LE OR SMOFlipid OR FMOS OR FMOS lipid OR Omegaven OR lipidem” and “(soybean oil OR plant oils OR fatty acids, omega-6 OR soy based lipid emulsion? OR (soy? adj2 oil?) OR (soy? adj2 lipid?) OR (soy? adj2 emulsion?) OR (soy? adj2 fat?) OR (omega-6 adj2 fatty acid?) OR plant oil? OR vegetable oil? OR olive oil? OR coconut oil? OR intralipid OR lipofundin OR clinoleic OR linoleic acid OR arachidonic acid)”. We also searched clinical trials and ISRCTN to get more initial information and identify unpublished data. No restrictions were placed on the searches for language, population, or publication year.

### Eligibility criteria

The inclusion criteria follow: (1) patients were neonates with birth weight less than 1500 g or gestational age less than 32 weeks with mean/median birth weight less than 1500 g in the whole group; (2) the intervention was fish oil-containing lipid emulsions compared to fish oil-free lipid emulsions administrated by PN; (3) the PN began within 24 hours after birth and lasted at least 1 week; (4) the observation outcome included the number of patients with cholestasis; and (5) study design was a randomized controlled trial (RCT).

The exclusion criteria follow: (1) patients with metabolic disorders, congenital infection, congenital abnormality, severe sepsis, or post-surgery; (2) birth weight or gestational age was not clear; (3) studies lacked initial clinical data, such as reviews, meta-analyses, comments, and editorials; and (4) observational studies.

### Data extraction

Two authors extracted the data independently from the full-text articles of all included studies. The disagreements were resolved by discussion or by consulting a third assessor. The following data were extracted: first author, publication year, study location, study design, birth weight of participants, cholestasis definition, sample size, intervention (type of lipid emulsion), PN duration, and the number of patients with cholestasis.

### Quality assessment

Two authors assessed the quality of the included studies using the Cochrane Risk of Bias tool for RCTs. This tool contains seven domains: sequence generation (selection bias), allocation concealment (selection bias), blinding of participants and personnel (performance bias), blinding of outcome assessment (detection bias), incomplete outcome data (attrition bias), selective reporting (reporting bias), and any other bias.

### Data analysis

Review Manager 5.3 was used to analyze the results. Because the outcome was a dichotomous value, a risk ratio (RR) and the corresponding 95% confidence interval (CI) were calculated. Heterogeneity was assessed using the *I*^2^ measurement, with *I*^2^ > 50% indicating significant heterogeneity. A fixed-effect model was used to summarize the data when *I*^2^ < 50% and a random-effect model was used when *I*^2^ > 50%. The subgroup analysis was performed to seek the source of heterogeneity. A sensitivity analysis was performed to evaluate the robustness of the conclusion by removing each study sequentially and examining the effect of removing each study on the pooled RR results. A funnel plot was constructed to detect reporting bias.

## Results

### Study selection

**Fig. 1:**
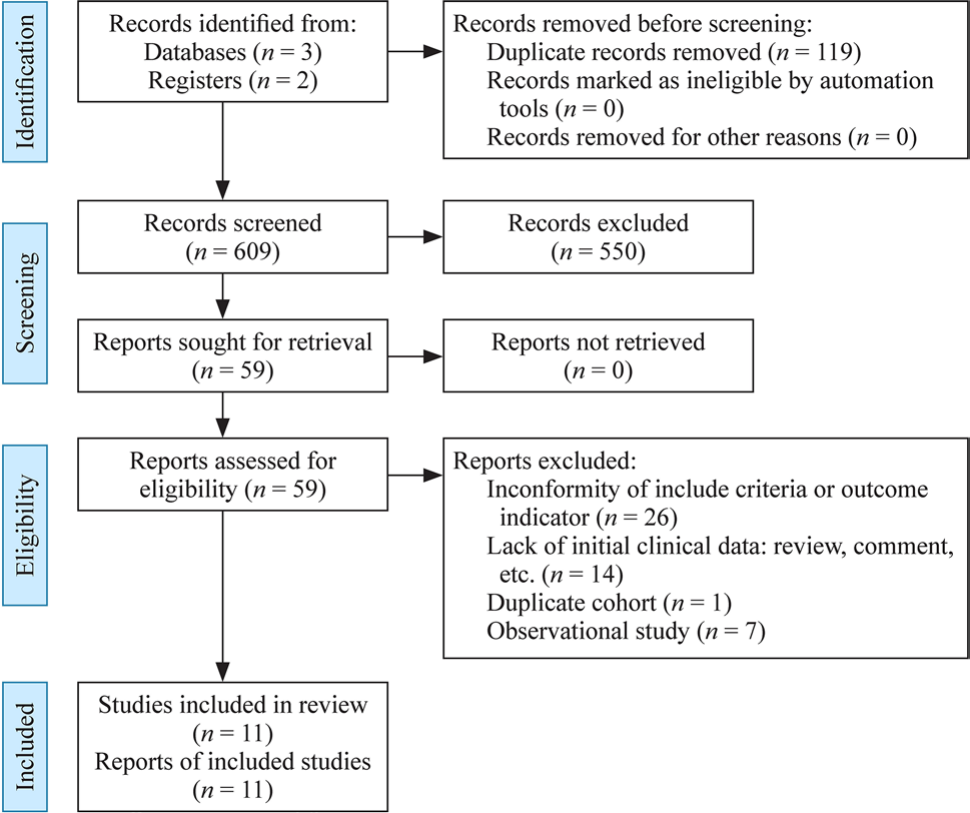
Flow-diagram of study screening

A total of 728 articles were retrieved, 119 of which were duplicated. Of the 609 articles, 550 were excluded on the basis of the title or abstract. Of the remaining 59 studies, 26 had inconformity of birth weight or gestational week or outcome indicator, 14 were reviews or comments which lacked initial data, one used a duplicated cohort, seven were observational trials, and 11 RCTs fulfilled the inclusion criteria and were used for the meta-analysis (Fig. 1). Of the 11 RCTs [19–29], three came from Turkey, two from Italy, one from Taiwan of China, one from Sweden, one from Poland, one from Austria, one from Greece, and one from the Netherlands. Three studies fixed the participants with gestational age under 32 weeks, but the mean/median birth weight was less than 1500 g. The remaining studies fixed the participants with birth weight less than 1500 g. One study did not clarify the definition of cholestasis but the others did. The interventions used fish oil-containing lipid emulsion and nine of which were SMOFlipid® (Fresenius Kabi, containing 30% soybean oil, 30% medium-chain triglycerides or MCT, 25% olive oil and 15% fish oil), one was mixed with 50% Omegaven® (Fresenius Kabi, containing 100% fish oil) and 50% ClinOleic® (Baxter spa, containing 80% olive oil and 20% soybean oil), and one was SMOFlipid® and Lipidem® (B. Braun, containing 50% medium-chain triglycerides, 40% soybean oil, and 10% fish oil). The control groups were fish oil-free lipid emulsions, four of which were Intralipid® (Fresenius Kabi, 100% soybean oil), five were ClinOleic®, one was Lipovenoes® (Fresenius Kabi, containing 50% medium-chain triglycerides and 50% soybean oil), and one was divided into three subgroups of Intralipid, Lipofudin® (B. Braun, containing 50% medium-chain triglycerides and 50% soybean oil), and ClinOleic®. Eight studies had mean/median parenteral duration more than 14 days, while three studies had mean/median parenteral duration of less than 14 days. The general characteristics are presented in Table 1.

**Table 1:** General characteristics of the included studies

Study ID	Location	Study design	Birth weight (g; I/C), mean ± SD or median (range)	Definition of cholestasis	Sample (I/C), *n*	Intervention (I/C)	PN duration (d; I/C), mean ± SD or median (range)	Cholestasis (I/C), *n*
Beken et al., 2014 [19]	Turkey	RCT	1092 ± 224/1160 ± 251	TB < 5 mg/dL, DB ≥ 1 mg/dL TB > 5 mg/mL, DB > 20% TB	40/40	SMOF®/Intralipid®	14 (10–28)/14 (10–21)	2/2
D'Ascenzo et al., 2014 [20]	Italy	RCT	909 ± 189, 888 ± 245/946 ± 197, 936 ± 225	DB > 2 mg/dL	39/41	SMOF®/Intralipid®	18/18	1/4
Hsiao et al., 2019 [21]	Taiwan of China	RCT	1004 ± 265/962 ± 194	DB > 2 mg/dL	30/30	SMOF®/50% MCT + 50% soybean oil	32.59 ± 16.84/31.62 ± 17.56	2/4
Najm et al., 2017 [22]	Sweden	RCT	799 ± 225/799 ± 225	DB > 50 μmol/L	41/37	SMOF®/ClinOleic®	12 (2–92)/12 (2–72)	4/2
Ozkan et al., 2019 [23]	Turkey	RCT	1397 ± 602/1302 ± 494	TB < 5 mg/dL, DB > 1 mg/dL TB > 5 mg/mL, DB > 20% TB	42/47	SMOF®/ClinOleic®	13.1 ± 2.2/13.1 ± 2.7	1/2
Pawlik et al., 2014 [24]	Poland	RCT	930 (580–1250)/940 (650–1250)	TB < 5 mg/dL, DB > 1 mg/dL TB > 5 mg/mL, DB > 20% TB	60/70	50% Omegaven® + 50% ClinOleic®/ClinOleic®	21.7 (5–55)/22.3 (7–91)	3/20
Repa et al., 2018 [25]	Austria	RCT	788 (648–891)/760 (610–884)	DB > 1.5 mg/dL	110/113	SMOF®/Intralipid®	23 (17–37)/24 (17–35)	11/18
Savini et al., 2013 [26]	Italy	RCT	898 ± 199, 93 ± 202/955 ± 202, 905 ± 160	DB > 2 mg/dL	55/89	SMOF® + Lipidem®/Intralipid® + Lipofundin® + ClinOleic®	20.9 ± 5.5, 19.0 ± 4.3/21.6 ± 5.6, 21.7 ± 6.9, 21.6 ± 6.5	2/1
Skouroliakou et al., 2016 [27]	Greece	RCT	1331 ± 290/1271 ± 199	Not reported	25/26	SMOF®/Intralipid®	> 15/> 15	4/3
Vlaardingerbroek et al., 2014 [28]	Netherlands	RCT	855 ± 266/888 ± 204	DB > 20% TB	48/48	SMOF®/ClinOleic®	11 (IQR 9–14)/12 (IQR 9–16)	2/2
Yildizdas et al., 2019 [29]	Turkey	RCT	1179 ± 440/1291 ± 446	TB < 5 mg/dL, DB > 1 mg/dL TB > 5 mg/mL, DB > 20% TB	34/33	SMOF®/ClinOleic®	25.2 ± 20/27.4 ± 16	0/6

*RCT* randomized control trials, *I* intervention group, *C* control group, *PN* parenteral nutrition, *TB* total bilirubin, *DB* direct bilirubin, *SD* standard deviation, *IQR* interquartile range, *SMOF®* Fresenius Kabi, containing 30% soybean oil, 30% medium-chain triglycerides or MCT, 25% olive oil and 15% fish oil, *Omegaven®* Fresenius Kabi, containing 100% fish oil, *ClinOleic®* Baxter spa, containing 80% olive oil and 20% soybean oil, *Lipidem®* B. Braun, containing 50% medium-chain triglycerides, 40% soybean oil, and 10% fish oil, *Intralipid®* Fresenius Kabi, 100% soybean oil, *Lipovenoes®* Fresenius Kabi, containing 50% medium-chain triglycerides and 50% soybean oil, *Lipofudin®* B. Braun, containing 50% medium-chain triglycerides and 50% soybean oil

### Quality of the included studies

**Fig. 2:**
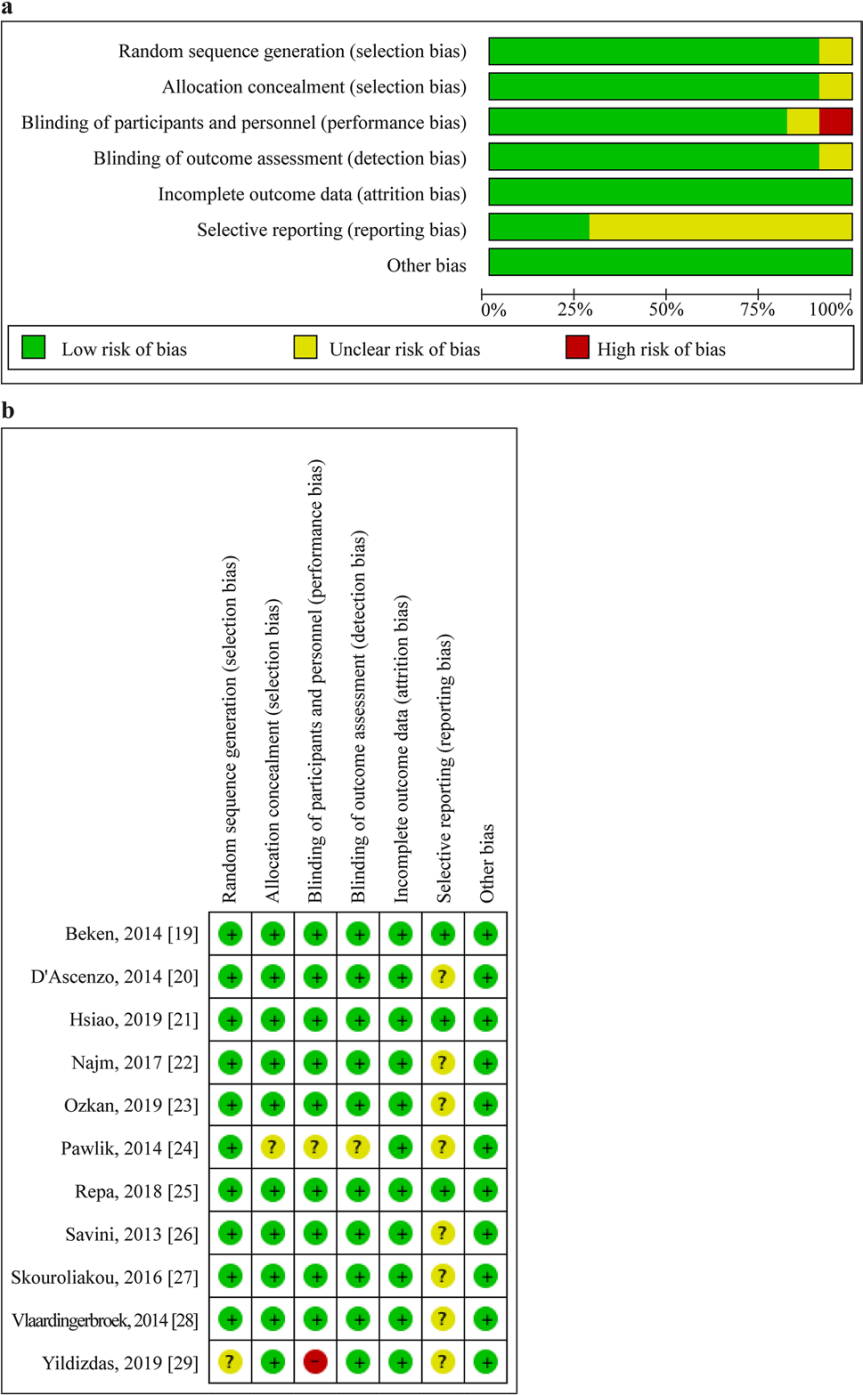
Quality of the 11 randomized controlled trials assessed using the Cochrane Risk of Bias tool.** a** Risk of bias graph for included studies; **b** risk of bias summary for included studies

Quality of the 11 RCTs assessed using the Cochrane Risk of Bias tool is shown in Fig. 2a and b. Of the 11 studies, one had a high risk of bias, three had a low risk of bias, and the remaining had unclear risk of bias.

### Occurrence of parenteral nutrition-associated cholestasis

**Fig. 3:**
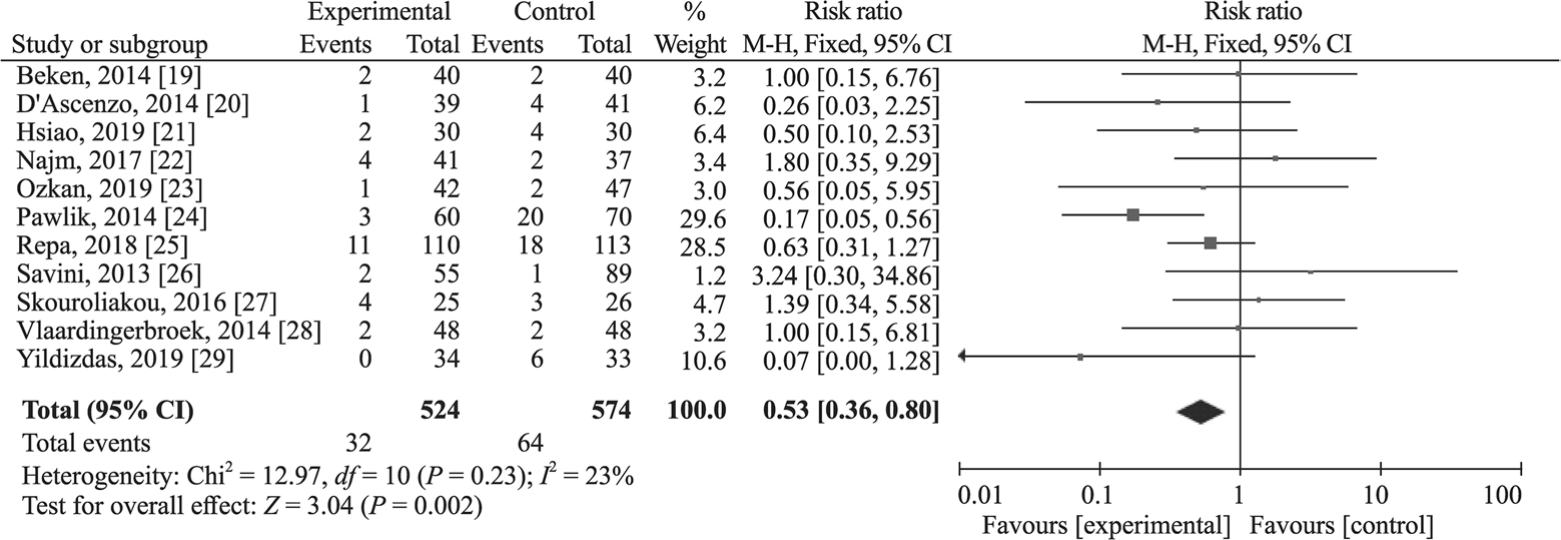
Meta-synthesis result of cholestasis occurrence for fish-oil containing vs. fish-oil free lipid emulsions. *CI* confidence interval

The 11 RCTs [19–29] included 524 infants in fish oil-containing lipid emulsion groups and 574 in fish oil-free lipid emulsion groups. Because heterogeneity was non-significant, with *I*^2^ = 23% (*P* = 0.23), a fixed-effect was used to synthesize the data. The result indicated that fish oil-containing lipid emulsion significantly reduced the PNAC occurrence with RR = 0.53, 95% CI 0.36–0.80, *P* = 0.002 (Fig. 3).

### Subgroup analysis

**Fig. 4:**
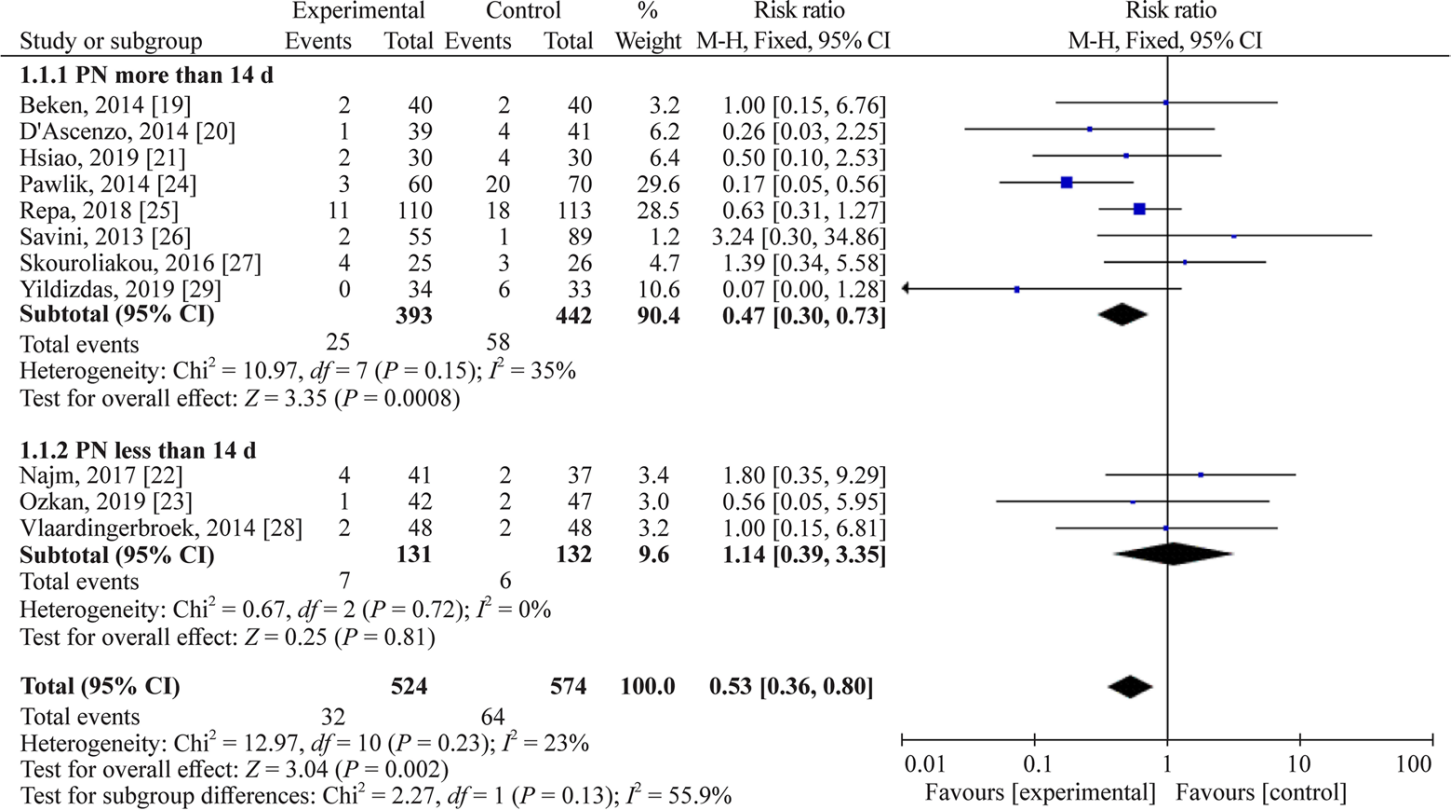
Subgroup analysis based on parenteral nutrition duration. *CI* confidence interval

**Fig. 5:**
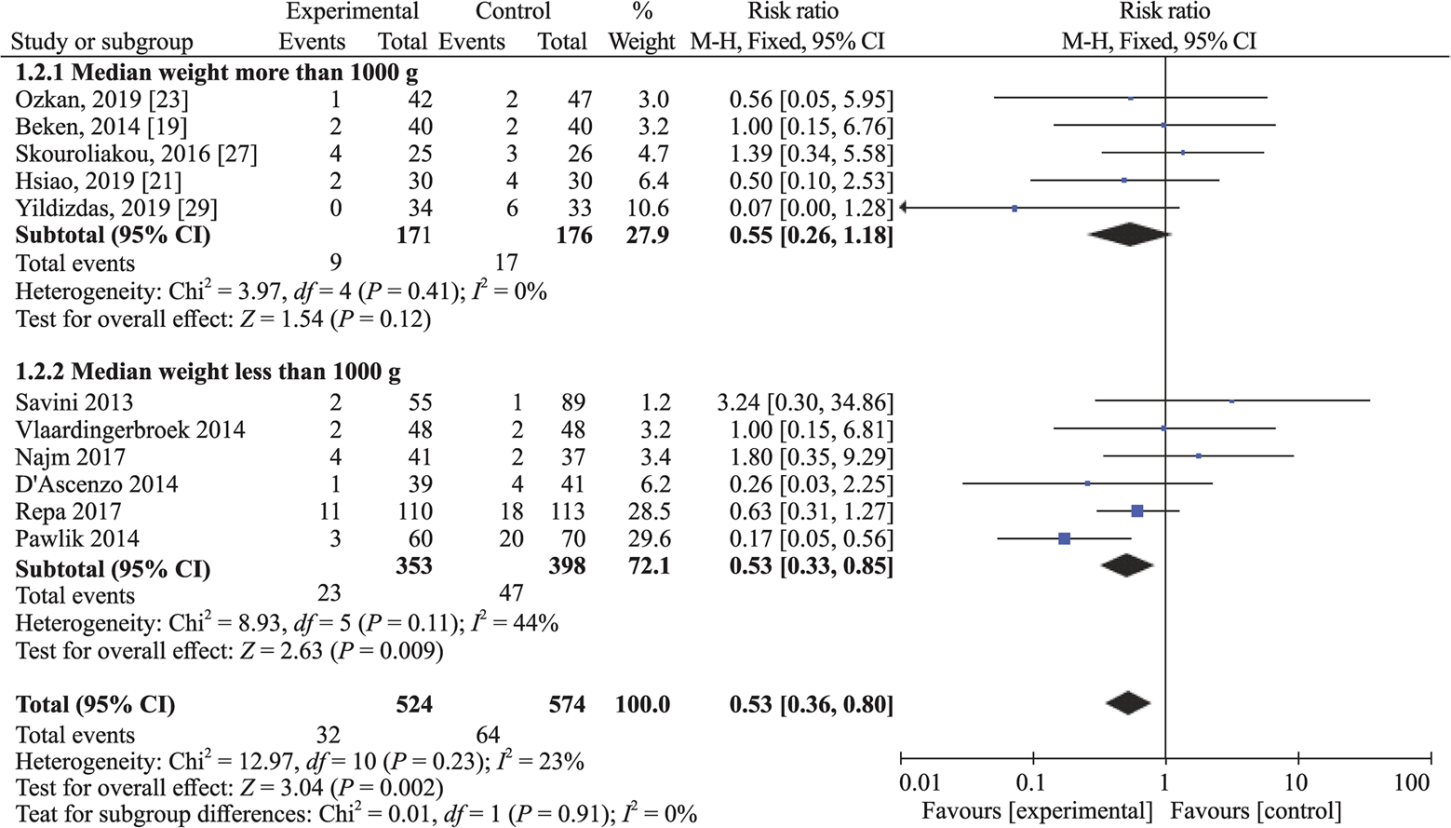
Subgroup analysis based on birth weight. *CI* confidence interval

Subgroup analysis based on PN duration and median birth weight was performed to analyze the source of heterogeneity. The heterogeneity analysis for patients with PN duration more than 14 days revealed *I*^2^ = 35% (*P* = 0.15) and pooled RR = 0.47, 95% CI 0.30–0.73, *P* = 0.0008; and patients with PN duration less than 14 days had *I*^2^ = 0% (*P* = 0.72) and pooled RR = 1.14, 95% CI 0.39–3.35, *P* = 0.81. The heterogeneity analysis for patients with birth weight more than 1000 g revealed *I*^2^ = 0% (*P* = 0.41) and pooled RR = 0.55, 95% CI 0.26–1.18, *P* = 0.12; and patients with birth weight less than 1000 g had *I*^2^ = 44% (*P* = 0.11) and pooled RR = 0.53, 95% CI 0.33–0.85, *P* = 0.009. Thus, the heterogeneity may come from studies of PN duration of more than 2 weeks and studies of median birth weight less than 1000 g (Figs. 4 and 5).

### Sensitivity analysis

Sensitivity analysis was performed by removing each study in turn. The result indicated that the study of Pawlik et al. [24] may be the source of heterogeneity, as *I*^2^ decreased to 0% and the synthesis result changed when it was removed. The total synthesis result was not influenced by removing any other study (Table 2).

**Table 2:** Sensitivity analysis results by removing each study

Removal study	Heterogeneity analysis			Pooled results		
	*χ*^2^	*I*^2^ (%)	*P*	RR (95% CI)	*Z*	*P*
Beken et al., 2014 [19]	12.69	29	0.18	0.52 (0.34, 0.78)	3.11	0.002
D’Ascenzo et al., 2014 [20]	12.36	27	0.19	0.55 (0.37, 0.83)	2.83	0.005
Hsiao et al., 2019 [21]	12.94	30	0.17	0.54 (0.35, 0.81)	2.93	0.003
Najm et al., 2017 [22]	11.15	19	0.27	0.49 (0.32, 0.75)	3.31	0.0009
Ozkan et al., 2019 [23]	12.98	31	0.16	0.53 (0.35, 0.80)	3.01	0.003
Pawlik et al., 2014 [24]	7.61	0	0.57	0.68 (0.44, 1.07)	1.68	0.090
Repa et al., 2018 [25]	13.04	31	0.16	0.50 (0.30, 0.81)	2.78	0.005
Savini et al., 2013 [26]	11.03	18	0.27	0.50 (0.33, 0.76)	3.26	0.001
Skouroliakou et al., 2016 [27]	11.42	21	0.25	0.49 (0.32, 0.75)	3.26	0.001
Vlaardingerbroek et al., 2014 [28]	12.70	29	0.18	0.52 (0.34, 0.78)	3.11	0.002
Yildizdas et al., 2019 [29]	10.61	15	0.30	0.59 (0.39, 0.89)	2.52	0.010

*RR* risk ratio, *CI* confidence interval

### Publication bias

**Fig. 6:**
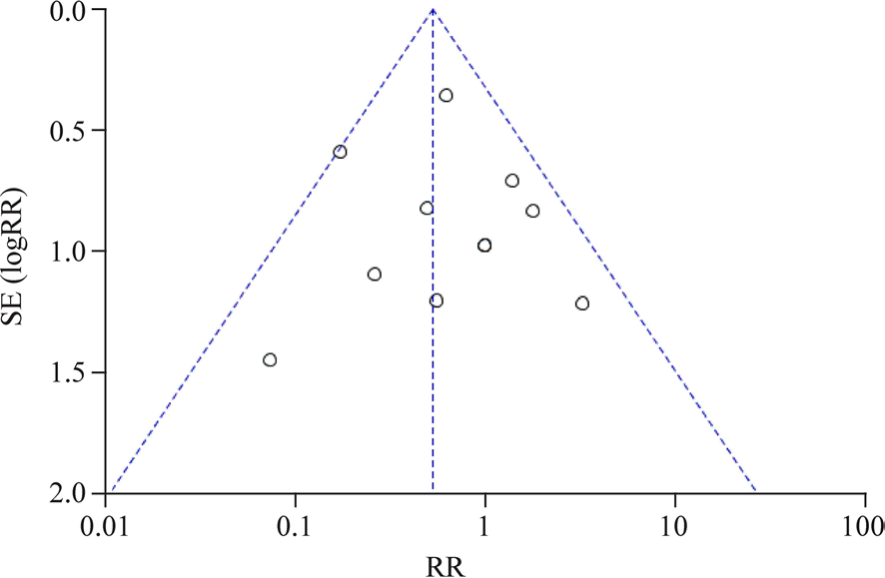
Funnel plot for included studies. *SE* standard error, *RR* risk ratio

A funnel plot was used to evaluate the publication bias. This indicated that publication bias was low, because more than 95% of studies were in the funnel and the plot was almost symmetric (Fig. 6).

## Discussion

The meta-analysis result revealed that fish oil-containing lipid emulsion significantly reduced the occurrence of PNAC by an average of 47% (range 20–64%, *P* = 0.002) in VLBW infants compared to fish oil-free lipid emulsions. For patients with PN duration exceeding 14 days, the preventive effect was significant, with an average reduction of 53% (range 27–70%, *P* = 0.0008), while it was non-significant for patients with PN duration less than 14 days (*P* = 0.81). The preventive effect was also remarkable in patients with birth weight less than 1000 g (i.e., ELBW infants), with an average reduction of 47% (range 15–67%, *P* = 0.009), but non-significant for those with birth weight exceeding 1000 g (*P* = 0.12). The meta-analysis had low to median heterogeneity. The subgroup analysis showed that the duration of lipid emulsion utilization and birth weight may be the sources of heterogeneity. The sensitivity analysis indicated that the study of Pawlik et al. [24] influenced the final result. The study of Pawlik et al. [24] showed a large effect estimate of fish oil lipid emulsion in reducing cholestasis (3/60 in experimental group versus 20/70 in control group). However, their study had unclear risk of bias according to the quality assessment. The allocation concealment and the blinding performance were not mentioned, which may lead to over-estimation for experimental group. The death rate was high (20/87 in experimental group and 18/88 in control group) and the withdrawal was unbalanced (7/87 in experimental group and 0/88 in control group), which may also influence the final result. Because this single study with an unclear risk of bias had a decisive role in the meta-syntheses, we should treat the final results with caution.

Because a longer duration of PN is needed for smaller and more premature infants, PNAC remarkably endangers VLBW infants. Kapoor et al. [30] and Park et al. [18] conducted meta-analyses to evaluate the overall preventive effect of fish oil-containing lipid emulsions on PNAC several years ago. The results showed no difference between intervention and control groups. However, their sample sizes were insufficient to draw an objective conclusion. Another meta-analysis in 2019 of Kapoor et al. [31] evaluated the effect of fish oil on PN-associated liver disease and cholestasis together, and gave similar results to ours. However, they did not focus on VLBW and ELBW infants. More recently, original studies including observational studies [32–39] and RCTs [19–29] have begun to focus on the prevention effect of fish oil-containing lipid emulsions on VLBW infants, but their results vary. Our meta-analysis provides an objective argument that fish-oil containing lipid emulsions can decrease occurrence of PNAC in VLBW infants.

Low birth weight and prolonged duration of PN are the major risk factors for PNAC [40]. In the subgroup analysis, fish oil-containing lipid emulsion had significant benefits for patients with PN duration exceeding 14 days and ELBW, indicating that fish oil-containing lipid emulsion could be a promising alternative lipid for ELBW infants. However, the PN durations in the studies were not all total PN durations. The different enteral feeding starting times and milk types during the transition from PN to enteral nutrition may influence the occurrence of cholestasis, and was not estimable in this study. In addition, medium-chain triglycerides, which are rapidly metabolized with high bioavailability and display a protective role in animal models of liver injury with anti-inflammatory effects [41, 42], may reverse or decrease the occurrence of cholestasis in neonates. In our meta-analysis, the lipid emulsions of intervention groups in 10 studies contained medium-chain triglycerides and only two studies had control groups containing medium-chain triglycerides, so the exact influence of medium-chain triglycerides cannot be evaluated either. Thus, it is difficult to determine the exact effect of different components of fish oil-containing lipid emulsion in preventing PNAC. Among the included studies, that of Pawlik et al. [24] had the most obvious preventive effect. The intervention group of this study used 50% Omegaven® (fish oil concentration 10%) and 50% ClinOleic®, equal to 5% of fish oil concentration in the whole lipid emulsion. The concentration of fish oil was higher than in any other groups (fish oil concentration in SMOF® 3% and Lipidem® 2%) and may be the reason for the best effect. Thus, the most suitable concentration of fish oil in the lipid emulsions is another question to be answered.

In addition to the problems mentioned above, the limitations of the study include that the sample size was small, with participant numbers in single studies ranging between 51 and 213, and the total number of patients in the intervention group was 524 and 574 in the control group for this specific meta-analysis. The heterogeneity among the studies is also unresolved. More large-scale, multi-center RCT, and homogenous studies could improve the reliability of any meta-analysis.

The meta-analysis updated the evaluation of the preventive effect of fish oil-containing lipid emulsion on PNAC in VLBW infants based on the available original RCT studies. The fish oil-containing lipid emulsion reduced the occurrence of cholestasis in VLBW infants by 47% on average (range 20–64%). Infants with PN duration exceeding 14 days and ELBW benefited more. The meta-analysis results supply new evidence for clinical workers to choose more suitable lipid emulsions for VLBW infants, especially ELBW infants. Future studies, focusing on the preventive effect of fish oil-containing lipid emulsion on total PN and comparing different single components and fish oil concentrations, will give a more robust conclusion.

## Data Availability

The data sets analyzed during the current study are available from the corresponding author on reasonable request.
